# Phase Segmentation Methods for an Automatic Surgical Workflow Analysis

**DOI:** 10.1155/2017/1985796

**Published:** 2017-03-19

**Authors:** Dinh Tuan Tran, Ryuhei Sakurai, Hirotake Yamazoe, Joo-Ho Lee

**Affiliations:** ^1^Graduate School of Information Science and Engineering, Ritsumeikan University, Kusatsu, Shiga, Japan; ^2^College of Information Science and Engineering, Ritsumeikan University, Kusatsu, Shiga, Japan

## Abstract

In this paper, we present robust methods for automatically segmenting phases in a specified surgical workflow by using latent Dirichlet allocation (LDA) and hidden Markov model (HMM) approaches. More specifically, our goal is to output an appropriate phase label for each given time point of a surgical workflow in an operating room. The fundamental idea behind our work lies in constructing an HMM based on observed values obtained via an LDA topic model covering optical flow motion features of general working contexts, including medical staff, equipment, and materials. We have an awareness of such working contexts by using multiple synchronized cameras to capture the surgical workflow. Further, we validate the robustness of our methods by conducting experiments involving up to 12 phases of surgical workflows with the average length of each surgical workflow being 12.8 minutes. The maximum average accuracy achieved after applying leave-one-out cross-validation was 84.4%, which we found to be a very promising result.

## 1. Introduction

In recent years, with advancements in technology and medicine, the operating room has evolved into a highly complex and technologically rich environment. Unfortunately, safety in such an environment remains not only unimproved and incommensurate with quality, but also a political issue. Errors are an inevitably integral part of human life, but errors in medicine are increasingly one of the most serious issues we face in daily practice. Although numerous research projects have focused on reducing medical errors and positive results have been achieved, there are still no radical solutions to stop such errors from occurring [1]. Aside from directly impacting patient safety, medication errors also significantly increase treatment costs for patients and their families. In the long term, they may affect patient mood as well as the mood of medical staff who made the errors. Causes for medication errors vary. In the operating room, although such errors can be caused by mistakes or failures of surgical equipment and devices, human mistakes are the most common. Examples here include procedural error, lack of documentation, lack of information of current state, miscommunication, lack of anatomical knowledge, and inexperience due to lack of training.

Moreover, in developed countries such as Japan, because the population is aging and declining, additional problems, such as a lack of medical staff, are occurring at increased rates in hospitals. To compensate for lack of staff, it is required to have either automated systems that can perform daily medical work in place of staff, or medical education support systems that can help to train larger numbers of staff in, among other areas, surgical techniques.

Many concepts and research topics have been proposed to solve the above medical problems using a step-by-step approach. In the operating room, patient safety system concepts, such as the context-aware operating room, which is able to monitor the safety of a surgical workflow in the operating room by having an awareness of all general working contexts within it, have been designed [2]. The context here can involve medical staff, patients, equipment, and medical materials. With its own awareness, context-aware patient safety systems are able to provide meaningful and important information, including current safety situations, and thus, if any unusual events do occur, the systems will issue warnings. Further, by using such a system, other processes are facilitated, including surgical decision-making and surgical training processes, thereby directly impacting patient safety. Such systems can also help in anticipating patient positioning, optimizing operating time, analyzing technical requirements, and the like.

To realize such a system, we first need to have an awareness of information regarding the current state of the surgical workflow. In general, if the system cannot detect the current state (i.e., phase), it will not be able to identify which surgical instruments should be used and what the staff must do in next phase; thus the system will not be able to issue any warnings and the decision-making process is severely hampered.

Because of the importance of such safety systems and in light of the growing interest in this field, in this paper, we propose new methods using latent Dirichlet allocation (LDA) and hidden Markov model (HMM) approaches to automatically segment the phases of a specified surgical workflow based on the motion features of the working contexts. Motion features are obtained by quantizing optical flow (OF) vectors extracted from videos captured by multiple synchronized cameras in the operating room. Note that LDA is a generative topic model that is widely used in natural language processing. Further, HMM is a statistical Markov model in which the system being modeled is assumed to be a Markov process with unobserved (i.e., hidden) states. Actually, in our previous work [3], surgical phase segmentation methods have been proposed. Although our previous work and this work have the same target: to output an appropriate phase label for each given time point of a surgical workflow in an operating room, the proposed methods in these works are almost different and the experiments are totally different.

In addition to this introductory section, this paper is organized as follows. In Section 2, we review related work. Details of our proposed methods are then described in Section 3. In Section 4, we present our experiments and results for evaluating the robustness of each method. Finally, we conclude our paper and provide avenues for future work in Section 5.

## 2. Related Work

Numerous methods have been developed for identifying intraoperative activities, segment common phases in a surgical workflow, and combine all gained knowledge into a model of the given workflows [4–7]. In segmentation work over surgical phases, various types of data were used, such as manual annotations by observers [8], sensor data obtained by surgical tracking tools based on frames of recorded videos [9, 10], intraoperative localization systems [4], and surgical robots [11]. In [4], Agarwal et al. incorporated patient monitoring systems used to acquire vital signals of patients during surgery. In [5], Stauder et al. proposed a method to utilize random decision forests to segment surgical workflow phases based on instrument usage data and other easily obtainable measurements.

Recently, decision forests have become a very versatile and popular tool in the field of medical image analysis. In [6], Suzuki et al. developed the Intelligent Operating Theater, which has a multichannel video recorder and is able to detect intraoperative incidents. This system is installed in the operating room and analyzes video files that capture surgical staff motions in the operating room. Intraoperative information is then transmitted to another room in real time to provide support for the surgical workflow via a supervisor. In [7], Padoy et al. used three-dimensional motion features to estimate human activities in environments including the operating room and production lines. They defined workflows as ordered groups of activities with different durations and temporal patterns. Three-dimensional motion data are obtained in real time using videos from multiple cameras. A recent methodological review of the literature is available in [12].

For medical terms, HMM has been used successfully in several research studies to model surgical activities for skill evaluation [13–15]. In [13], Leong et al. recorded six degrees-of-freedom (DOF) data from a laparoscopic simulator and then used them to train a four-state HMM to classify subjects according to their skill level. In [14], Rosen et al. constructed an HMM using data from two endoscopic tools, including such data as position, orientation, force, and torque. Here the HMM was able to identify differences in the skill levels of subjects with different levels of training. In [15], Bhatia et al. segmented four phases, namely, a patient entering or leaving the room, also the beginning and the end of a surgical workflow by using a combination of support vector machines (SVMs) and HMMs from video images.

## 3. Proposed Methods

**Figure 1:**
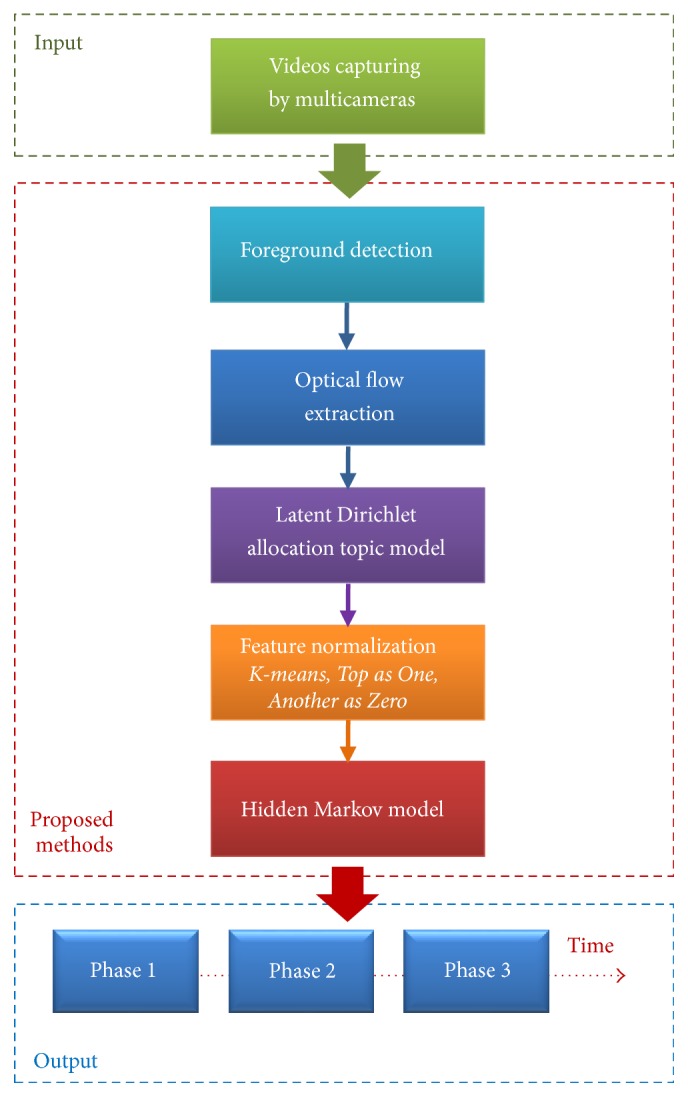
Overview of our proposed system.

As noted above, analyzing the surgical workflow, in particular, for surgical phase segmentation, is a research domain that has seen increased interest in context-aware operating room environments. The goal of our present work is to output an appropriate phase label for each given time point of multiple synchronized videos captured from a surgical workflow in an operating room. The term* phase* in our paper indicates a specified task or state in a surgical workflow. In each task of any surgical workflow, staff perform surgery by doing sequential actions. Therefore, using OF extraction to extract motions from these actions can provide effective features. Moreover, because each type of surgery has its own order of phases that must be done and there is no switching of this order between these phases, our approach is to construct an HMM based on this characteristic with the number of hidden states corresponding to the number of phases in the surgical workflow. The observed value of the HMM at a given time point will thus be calculated from normalized motion features learned via OF extraction and LDA after using a foreground detection method to reduce noise. The output likelihood of the HMM will indicate the appropriate phase corresponding to that time point.  Figure 1 shows the flow of our proposed system, with details explained in the following subsections.

### 3.1. Foreground Detection

Foreground detection is a crucial technique wherein a frame's foreground, for example, a person, an animal, and a vehicle, is detected before operating further complex processes for segmentation, recognition, tracking, and so forth. Like other computer vision tasks, after a frame preprocessing stage (such as a Gaussian blur filter to eliminate noise) is applied, foreground detection is applied in our work to extract the area that contains staff performing surgery, as well as the movements of surgical instruments or materials, namely, the region of interest (ROI) from the background of a frame sequence in the operating room.

Many FD methods have been designed, using such techniques as frame difference, mean filter to statistical methods using single or multiple-Gaussian models, statistical methods using color and texture features, fuzzy or neural networks methods, and methods based on eigenvalues and eigenvectors [16]. Each of these methods has its own benefits, but a robust method should be able to handle scene and condition changes caused by ambient lighting or nonstatic background objects. In [16], Sobral and Vacavant compared a wide range of state-of-the-art foreground detection methods. The* static frame difference* method, which uses a manually selected static background frame to compute the absolute difference between it and each video frame, is the simplest approach; however, a static frame is not the best choice because the background may change over time and therefore limit accuracy. Alternatively, we deem it better to use the* frame difference* approach, which uses the previous frame rather than a static frame to compute differences. By using this method, background changes can be resolved, but if the foreground object suddenly stops moving, the foreground detection process will fail. Therefore, more robust methods are needed to adapt to more complex environments.

**Figure 2:**
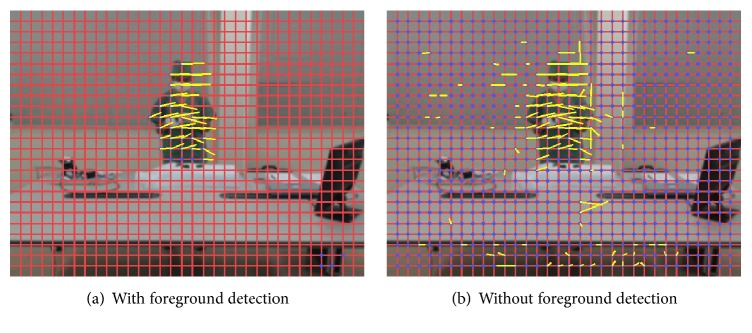
An example of extracted OFs with or without foreground detection.

**Figure 3:**
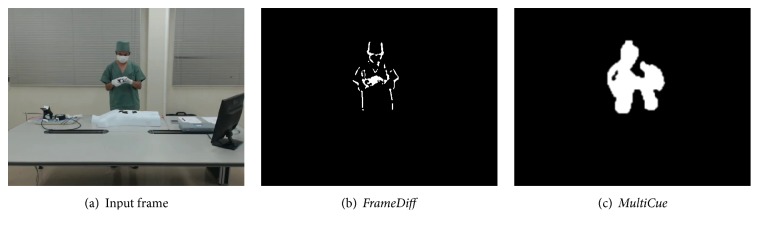
An example of an input frame and corresponding foreground detection results.

In our work, we used two methods, namely,* frame difference* and* multicue* [17], to detect the foreground and then obtain the ROI based on the characteristics in the detected areas and the differences between their execution times. By executing this step, we will ensure that only movements of staff, surgical instruments, and materials are monitored.  Figure 2 shows an example of extracted OFs in Section 3.2 with or without foreground detection. The figure indicates that OFs which were extracted without foreground detection include a lot of noise, while OFs which were extracted after detecting foreground have very less noise.  Table 1 shows all applicable methods with respective authors, method names, and abbreviations, the latter arbitrarily denoted for convenience. Further, Figure 3 shows an example input frame and the difference between the detection results obtained via the two methods. We observe that* FrameDiff* is only able to detect the contour of the moving staff, whereas* MultiCue* tends to also detect the region inside the contour.

### 3.2. Optical Flow Extraction

OF is the pattern of the apparent motion of objects, surfaces, and edges between two video frames with a small time interval. Used in a variety of studies [7, 18], OF extraction is used to extract OFs between two consecutive frames in a frame sequence. In our present work, we propose two methods for extracting motion features. The first method is called* grid intersection* OF extraction, while the other is called* grid block* OF extraction. For convenience, we use the abbreviations shown in Table 2 to refer to these methods.

#### 3.2.1. Grid Intersection OF Extraction

**Figure 4:**
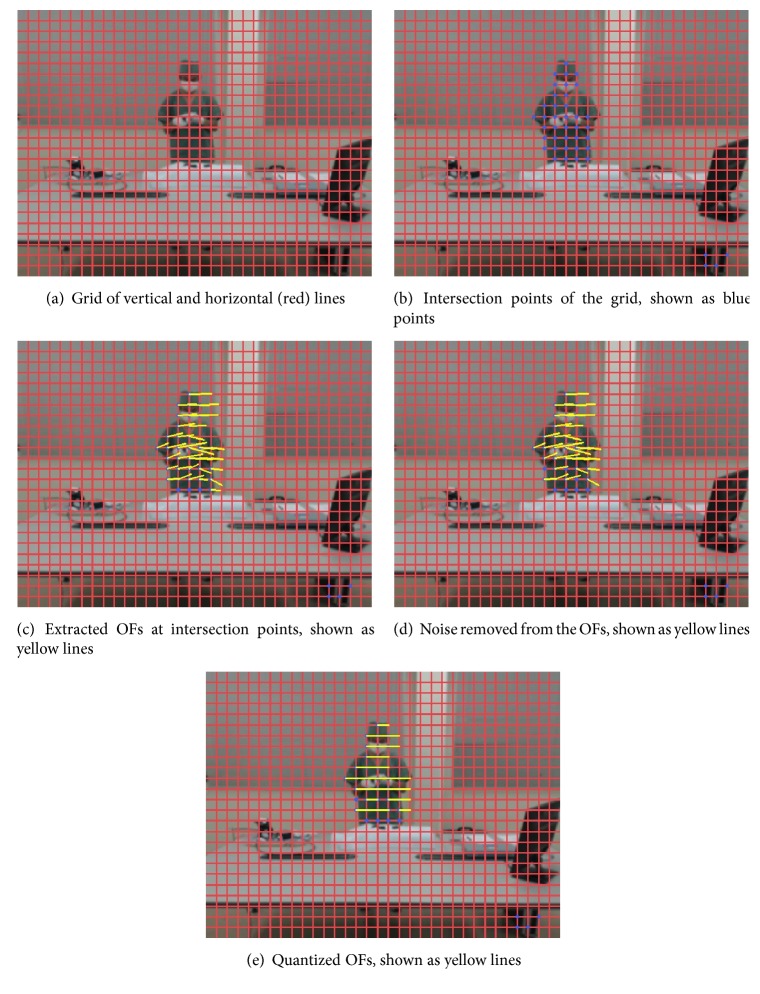
Example outputs of all steps in the* GridIntersect* method.

In this method, using synchronized videos acquired from multiple cameras, we extract OFs from each consecutive pair of Gaussian-blurred frames at points (i.e., pixels) arranged on a grid with a spacing of fixed pixels using the techniques described in [19]. First, all extracted OFs are restricted within a predefined range of minimum ($\overset{ˇ}{o}$) and maximum ($\hat{o}$) values of its own vector magnitude to eliminate noise. The remaining OFs are then normalized into four directions, namely, up, down, left, and right. After normalization is applied, all OFs have the same size.  Figure 4 shows an example of the outputs from all steps of this method, with all OF vectors in the figure having magnitudes 20 times larger than actual values. The final output is a set of OFs at all time points (i.e., seconds) in the surgical workflow videos at all grid intersection points (see Figure 6(a)).

#### 3.2.2. Grid Block OF Extraction

**Figure 5:**
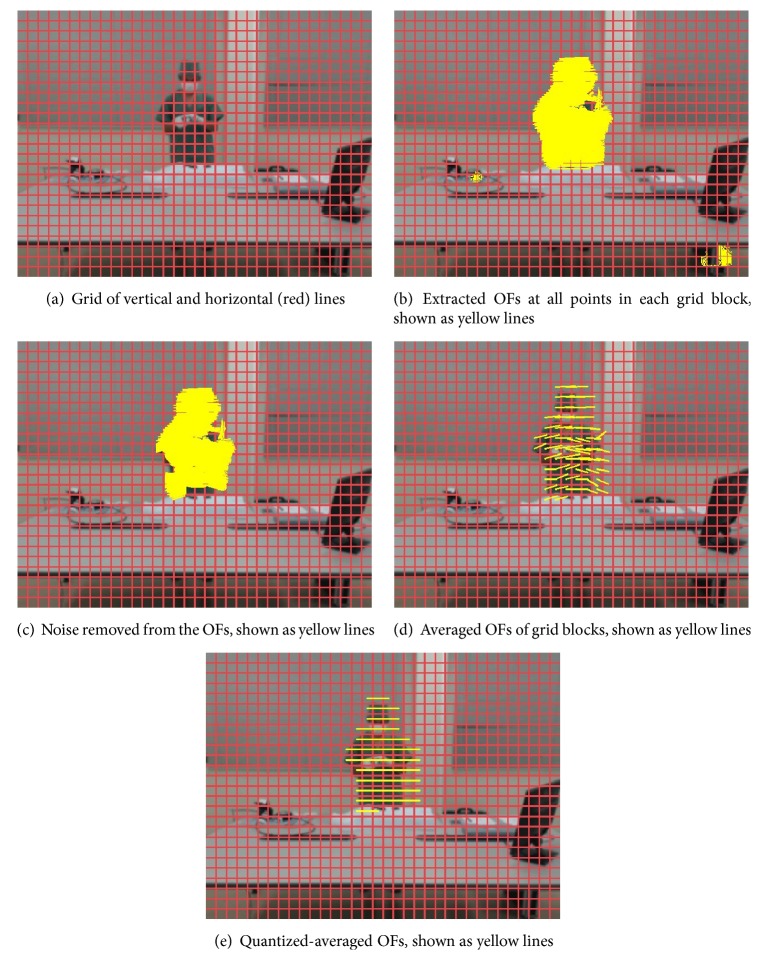
Example outputs of all steps in the* GridBlock* method.

**Figure 6:**
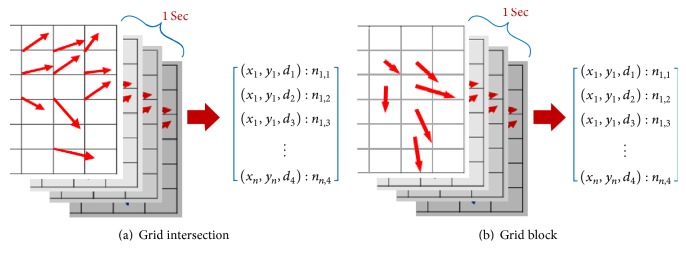
An example of OF extraction outputs. (*x*, *y*) denotes the coordinate of grid intersection point or block. *d* is one of four directions, namely, up, down, left, and right. *n* is the number of OFs acquired in one second.

After the frame preprocessing stage, for example, using a Gaussian blur filter to eliminate noise, vertical and horizontal lines, the same as the above* GridIntersect* method, are used to divide each frame into a grid of equal blocks; however, in the* GridBlock* method, OFs at all points in each block are extracted rather than at the intersection points between lines. In particular, from all extracted OFs, we ignore OFs that have own magnitudes outside of the given threshold range ($\overset{ˇ}{o}$ and $\hat{o}$). Next, we count the remaining OFs in each block to ignore blocks with the number of OFs inside smaller than minimum threshold value $\ddot{o}$. We then calculate an average OF from the OFs in every remaining block and then finally normalize along the four directions, just as with the* GridIntersect* method. An example of the outputs from all steps is shown in Figure 5, with all OF vectors having magnitudes 20 times larger than actual values. The final output of the* GridBlock* method is a set of OFs at all time points (i.e., seconds) in the surgical workflow videos at all grid blocks (see Figure 6(b)).

### 3.3. Foreground Detection and OF Extraction Combinations

As mentioned in Section 3.1, in this work, we use the two methods shown in Table 1 to detect the foreground of the surgical workflow videos in the operating room. Using these methods, OFs extracted at points not in the foreground are ignored. Here, by combining the two foreground detection options with the two OF extraction methods, as described in Section 3.2, we construct a total of four methods to extract motion features, as summarized in Table 3.

### 3.4. OF Quantization

After extracting OFs between each pair of consecutive frames, all OFs are quantized in terms of* words*, which will be used in Section 3.5. A word here indicates a normalized OF direction at a specified position, namely, either a grid intersection point or grid block. To see this, we assume that the number of synchronized cameras *ς* is two, each frame is divided into a grid of 10 × 10 intersection points or 10 × 10 blocks, and the number of normalized OF directions is four (i.e., up, down, left, and right). The number of words in the corpus in this case, also known as* vocabulary size*, is therefore 2 × 10 × 10 × 4 or 800.

### 3.5. Latent Dirichlet Allocation (LDA) Topic Model

**Figure 7:**
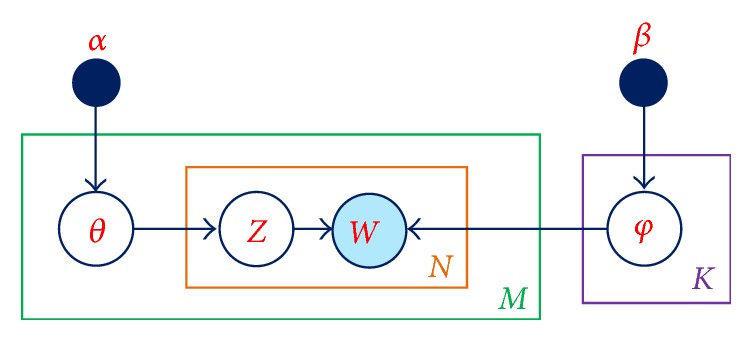
LDA graphical model.

As described in [20], LDA is a generative model widely used in natural language processing. As with other statistical models, topic models such as LDA use statistical inferences to learn topics that appear inside documents of a corpus. In LDA, each document can be understood simply as a set of words with its own ratio of all topics assumed to be inside it. The graphical model of LDA is shown in Figure 7. Here, *M* represents the number of documents, *N* denotes the number of words in a document, and *K* represents the number of topics. Further, *α* is the parameter of the Dirichlet prior on the per-document topic distributions, *β* is the parameter of the Dirichlet prior on the per-topic word distributions, *θ*_*i*_ is the topic distribution for document *i*, *ϕ*_*k*_ is the word distribution for topic *k*, *z*_*i*,*j*_ is the topic for the *j*th word in document *i*, and *w*_*i*,*j*_ represents a specific word.

Although LDA,* document*, and* word* are well-known in natural language processing, these terms are abstract here and should not be limited to normal text documents; as such, these terms can also be applied to other kinds of data, including images and speech. In our present work, LDA is used to infer topics that appear inside each time point of a surgical workflow; these inferences are based on the motion features represented by OFs extracted from the movements of staff and any surgical instruments or materials in the operating room. The main purpose of using LDA in this work is to learn not only the appearance of OFs, but also the dependencies among them. Without LDA, OFs which were extracted in Section 3.4 can be used directly as the input of one of feature normalization methods in Section 3.6. Unfortunately, in this way, only the appearance of OFs is covered; the dependencies among them are omitted.

**Figure 8:**
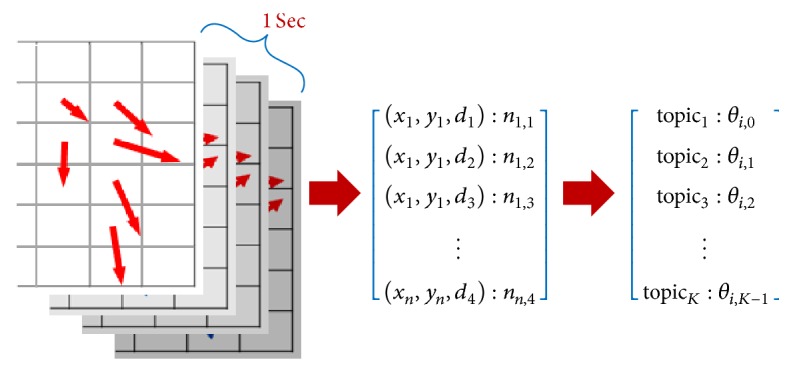
OF extraction output and LDA topic distribution for *i*th one-second clip. (*x*, *y*) denotes the coordinate of grid intersection point or block. *d* is one of four directions, namely, up, down, left, and right. *n* is the number of OFs acquired in one second. *θ*_*i*,*j*_ is the ratio of *j*th topic in *i*th document. *K* is the number of LDA topics.

First, all synchronized videos captured by multiple cameras are divided into a sequence of *ς* one-second clips, where *ς* is the number of synchronized cameras. Indeed, each clip corresponds to a document in LDA, while each document is represented by the words accumulated over its entire set of frames (see Section 3.4). The final output of LDA is shown in Figure 8, which is the distribution of all topics over each one-second clip, which we present as (1) below, and the distribution of all words over each topic, presented as (2) below.

(1) θi~Dirα,i0,…,M−1,

(2) ϕk~Dirβ,k0,…,K−1.

### 3.6. Feature Normalization

As explained in Section 3.1 above, the output of LDA is the distribution of all topics over all *ς* one-second clips (i.e., documents), which is actually the ratio of all topics for each document. This output can be understood simply as the motion features learned by both OF extraction and LDA; thus we can represent each second of the surgical workflow by a *K*-dimensional feature vector, presented as follows:

(3) θi=θi,0,θi,1,…,θi,K−1,∑j=0K−1θi,j=1.

Here, *K* represents the number of topics.

Before training an HMM from all motion feature vectors of the training surgical workflow videos, we use a feature normalization method to translate these feature vectors into a more familiar range; we also construct a faster and more accurate HMM. We achieve this because we not only eliminate noise, but also facilitate faster convergence of the iterative parameter estimation method that is part of the HMM.

In this subsection, we therefore introduce two methods to normalize the features. While the first method is rather simple, called* Top as One, Another as Zero*, the second method uses a *k*-means approach, which is a* hard clustering* method to yield new feature vectors. Like other sections, for convenience, we denote the abbreviations for our methods as shown in Table 4. Equation (4) shows an example of the feature normalization output for a one-second clip (OF extraction output, LDA topic distribution, and normalized feature for *i*th one-second clip).

(4) $$\begin{array}{c} \left[\begin{array}{c} \left(x_{1},y_{1},d_{1}\right):n_{1,1}\\ \left(x_{1},y_{1},d_{2}\right):n_{1,2}\\ \left(x_{1},y_{1},d_{3}\right):n_{1,3}\\ \vdots \\ \left(x_{n},y_{n},d_{4}\right):n_{1,4} \end{array}\right]⟶\left[\begin{array}{c} {topic}_{1}:\theta_{i,0}\\ {topic}_{2}:\theta_{i,1}\\ {topic}_{3}:\theta_{i,2}\\ \vdots \\ {topic}_{K}:\theta_{i,K-1} \end{array}\right]⟶\left[\begin{array}{c} \vdots \\ 1\\ \vdots \\ 0\\ \vdots \\ 0\\ \vdots \end{array}\right], \end{array}$$

where (*x*, *y*) denotes the coordinate of grid intersection point or block. *d* is one of four directions, namely, up, down, left, and right. *n* is the number of OFs acquired in one second. *θ*_*i*,*j*_ is the ratio of *j*th topic in *i*th document. *K* is the number of LDA topics.

#### 3.6.1. Top as One, Another as Zero

In this method, each *K*-dimensional feature vector *θ*_*i*_ is assigned to a new binary-based *K*-dimensional vector, namely, *θ*_*i*_^*∗*^, in which only the dimension with the maximum value in the old vector takes on values of one, whereas the others are set to zero, summarized as follows:

(5) $$\begin{array}{c} \theta_{i,j}^{*}=\left\{\begin{array}{cc} 1, & if\;\;j=\arg\underset{\alpha }{\max}\left(\theta_{i,\alpha }\right),\\ 0, & otherwise, \end{array}\right.\;j,\alpha \in \left[0,K\right). \end{array}$$

#### 3.6.2. *K*-Means

*K*-means clustering is a method for vector classification that solves the well-known clustering problem in data mining. In unsupervised learning, *k*-means is one of the simplest algorithms in that its procedure follows a straightforward method for classifying a given set of *n* feature vectors into a certain number of clusters (i.e., *c* clusters) in which each feature vector belongs to the cluster with the nearest mean.

The main idea behind *k*-means is to define *c* centers, with each center corresponding to a cluster. Because the different locations of these centers cause different results, it is better to place them as far away from one another as possible. The first step is to associate each feature vector to the nearest center and then achieve an early group age. In the next step, the *c* centers must be recalculated based on the associated results from the previous step. After *c* new centers are generated, each feature vector is reassociated with the nearest new center. These actions are repeated until there are no more changes in the location of the *c* centers.

More specifically, given set *θ*_*i*_ of *K*-dimensional feature vectors, with *i* = 0,…, *n* − 1, *k*-means classifies the *n* feature vectors into *c* (≤*n*) clusters *S* = *S*_0_, *S*_2_,…, *S*_*c*−1_ by finding positions *μ*_*i*_, *i* = 0,…, *c* − 1, of the cluster centers that minimizes the within-cluster sum of squares, which serve as the distances from feature vectors to the clusters centers, namely,

(6) $$\begin{array}{c} \arg\underset{S}{\min}\overset{c}{\underset{i=1}{\sum }}\;\underset{\theta \in S_{i}}{\sum }{\left\|\theta -\mu_{i}\right\|}^{2}. \end{array}$$

Using this normalization method, instead of using (5), each *K*-dimensional feature vector *θ*_*i*_ is assigned to a new binary-based *c*-dimensional, *θ*_*i*_^*∗*^, which corresponds to *c* clusters, using (7) below. Only dimension *j* in *θ*_*i*,*j*_^*∗*^ that corresponds to index *α* of the nearest center *μ*_*α*_ is set to one, whereas the other values are set to zero.

(7) $$\begin{array}{c} \theta_{i,j}^{*}=\left\{\begin{array}{cc} 1, & if\;\;j=\arg\underset{\alpha }{\min}\left\|\theta_{i}-\mu_{\alpha }\right\|,\\ 0, & otherwise, \end{array}\right.\;j,\alpha \in \left[0,c\right). \end{array}$$

### 3.7. Hidden Markov Model (HMM)

**Figure 9:**
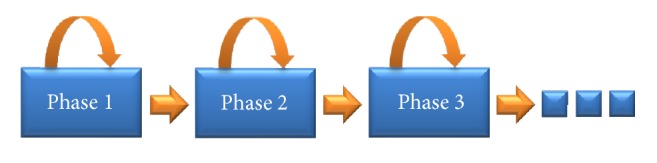
*Left to right* HMM graphical model.

The HMM approach is a statistical Markov model in which the system being modeled is assumed to be a Markov process with unobserved (i.e., hidden) states or phases [18]. Each state has a state transition probability distribution that defines the transition probabilities to other states, an emission probability distribution that defines output probabilities for all observed values in the state, and an initial state probability distribution that represents the probability that this state is the starting state of the HMM. Because each kind of surgery has its own order of phases that must be performed and there is no switching of the order of these phases, a* left to right* HMM is appropriate for our work here. A* left to right* HMM is limited in terms of transition probability distribution in that all states are able to transition to themselves or the next state and are not able to return to previous states (see Figure 9).

First, the transition probability from state *q* to state *q*′ is calculated via

(8) $$\begin{array}{c} T_{q,q^{\prime }}=\frac{N_{q\to q^{\prime }}}{N_{q\to -}}, \end{array}$$

where *N*_*q*→*q*′_ represents the number of transitions from state *q* to state *q*′ and − indicates all states. Because of the characteristics of the* left to right* HMM, the transition probability distribution can then be expressed as the following matrix:

(9) $$\begin{array}{c} \left[\begin{array}{cccccc} T_{0,0} & 1-T_{0,0} & 0 & \cdots & 0 & 0\\ 0 & T_{1,1} & 1-T_{1,1} & \cdots & 0 & 0\\ 0 & 0 & 0 & \cdots & T_{Q-2,Q-2} & 1-T_{Q-2,Q-2}\\ 0 & 0 & 0 & \cdots & 0 & 1 \end{array}\right] \end{array}$$

Here, *Q* represents the number of states in the HMM.

Second, the initial state probability distribution, which represents the probability that a state is the starting state of the HMM, is defined as

(10) $$\begin{array}{c} \pi_{q}=\left\{\begin{array}{cc} 1, & if\;\;q=0,\\ 0, & otherwise. \end{array}\right. \end{array}$$

Third, from the normalized feature vectors *θ*_*i*_^*∗*^, *i* = 0,…, *n* − 1, obtained in Section 3.6, the emission probability distribution of state *q* is calculated using

(11) $$\begin{array}{c} E_{q,\theta^{*}}=\frac{\sum_{i}\left(i\in q\;|\;\theta_{i}^{*}=\theta^{*}\right)}{\sum_{i}\left(i\in q\right)}+\epsilon . \end{array}$$

Here, ∑(*i* ∈ *q*) is the number of documents in state *q*, ∑(*i* ∈ *q*∣*θ*_*i*_^*∗*^ = *θ*^*∗*^) is the number of documents in state *q* with normalized feature vectors, also known as* observation value*  *θ*^*∗*^, and *ϵ* is a smoothing term. Note that ∑_*q*_∑_*i*_(*i* ∈ *q*) will simply be *n*, the number of documents in the surgical workflow.

The HMM is initialized with all parameters calculated above. These model parameters are then estimated with the goal of maximizing the likelihood of the data given the model. We accomplish this via the Baum-Welch algorithm [21], which is actually an instance of the well-known Expectation-Maximization algorithm for missing data problems in statistics [22, 23]. The process is iterative and hence we call it reestimation.

### 3.8. Phase Segmentation

After the HMM has been built from training videos captured by multiple synchronized cameras in the operating room for a specified surgical workflow, each test video of a surgical workflow for the same surgery type is divided into a sequence of one-second clips. Foreground detection, OF extraction, and OF quantization are applied in the same way as in the training process (see Sections 3.1, 3.2, 3.3, and 3.4). The distribution of all topics over each one-second clip is then calculated based on the quantized OFs using the topic model constructed in the training process (see Section 3.5). Next, each calculated distribution is normalized by using* Top* in Section 3.6.1 or *k*-means in Section 3.6.2 based on the estimated *c* cluster centers *μ*. Normalized feature of each one-second clip is finally used to estimate phase label for that clip based on probabilities in (8) and (11), which are calculated from the estimated HMM parameters in the training process (see Section 3.7).

## 4. Experiments

In this section, we describe experiments that we conducted to evaluate the performance of our proposed methods (Table 6). We first describe the general setting in which we performed the experiments and then present detailed results and a discussion in Section 4.2.

### 4.1. Experimental Settings

The surgical workflow used in our experiments was cholecystectomy, which is a typical laparoscopic surgery. This surgical workflow has a basic flow assumed to consist of the 12 phases shown in Table 5. Currently, it is difficult to prepare cameras in a hospital to record real surgical workflows due to human rights and privacy issues. Therefore, in this paper, we describe a simulation of the operating room that we constructed in our laboratory in which we recorded surgical workflow videos under conditions that were as similar to a realistic surgical workflow as possible. To accomplish this, we prepared real equipment, medical materials, including a laparoscope, which is an elongated rod with a miniature camera attached on the top of the rod to observe inside a patient's abdomen, monitors, trocars, forceps, and a carbon dioxide inhalator.

**Figure 10:**
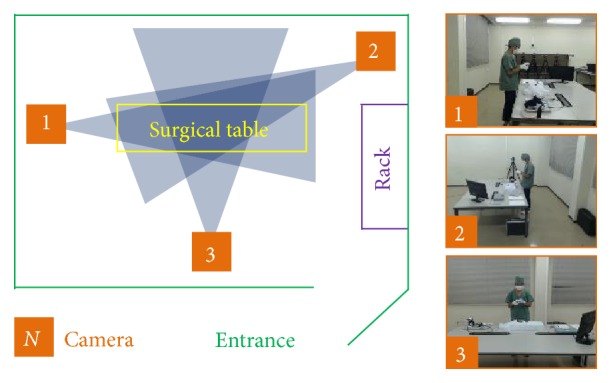
Simulated setting for our experiments, plus sample camera images.

Figure 10 shows the overall setting of our simulated operating room. We placed three cameras on three different walls far enough away from the operating area, which is marked “surgical table” in the figure. The cameras were synchronized by sending and receiving synchronization messages between them and a host computer. Therefore, despite the existence of cameras in the operating room, all staff, including surgeons, do not notice the presence of such cameras; thus there is no risk for the surgical workflow to be negatively impacted due to their existence. This is a substantial advantage as compared to other sensors. All cameras had a resolution of 640 × 480; the figure also shows example frames captured by the cameras.

We recorded the surgical workflow 28 times with seven different participants, each participant being recorded four times by the three different cameras; thus we obtained a total of 84 videos. The average length of surgical workflows was 12.8 minutes. The frame rate of all video cameras was 24 frames per second. The average number of frames of each video was then 18432. The participants in our experiments are normal people who imitate surgery operation. For each parameters setting, of the 28 recorded surgical workflows, 27 were used for training; the remaining surgical workflow recordings were used to test the accuracy of our proposed methods. We used this approach such that every set of videos was used for testing once in a leave-one-out cross-validation strategy. From this, all results shown in Section 4.2 are then the average accuracy after repeating this process 28 times and all parameter values were fixed. By using average accuracy to evaluate methods, we can find out which method works well in almost every surgical workflow. More specifically, the average accuracy of a method for a fixed parameters setting was calculated as follows: First, ratio of the amount of time in which phase label was estimated correctly and the true phase length in second unit was calculated in each phase of a test surgical workflow. Next, the average ratio of all phases in the test workflow was calculated. This is defined as the accuracy in a test workflow. Finally, the average accuracy was obtained by averaging all accuracies in all 28 test workflows. In addition, the true length for each phase in every surgical workflow was manually decided by us before constructing HMM, because the true phase length affects how HMM is constructed.

The size of the Gaussian filter used to smooth frames was 7 × 7, and the grid size related to the vertical and horizontal lines described in Section 3.2 was 64 × 48. We set parameters $\overset{ˇ}{o}$ and $\hat{o}$ to two and 20, respectively, while $\ddot{o}$ was set to five. From Section 3.5, the number of topics, *K*, was increased from 10 to 100 by a step of 10. Parameters *α* and *β* for the LDA were set to 50/*K* and 0.1, respectively. Smoothing term *ϵ* in (11) was set to 0.0001, and the number of clusters *c* for *k*-means from Section 3.6 ranged from 10 to 400 by a step of 10.

Moreover, to calculate average elapsed time for a test surgical workflow with the average workflow length being 12.8 minutes, we used a 2.30 GHz Intel Core i7-4712HQ central processing unit without graphics processing unit implementation.

### 4.2. Experimental Results

#### 4.2.1. Accuracy

**Figure 11:**
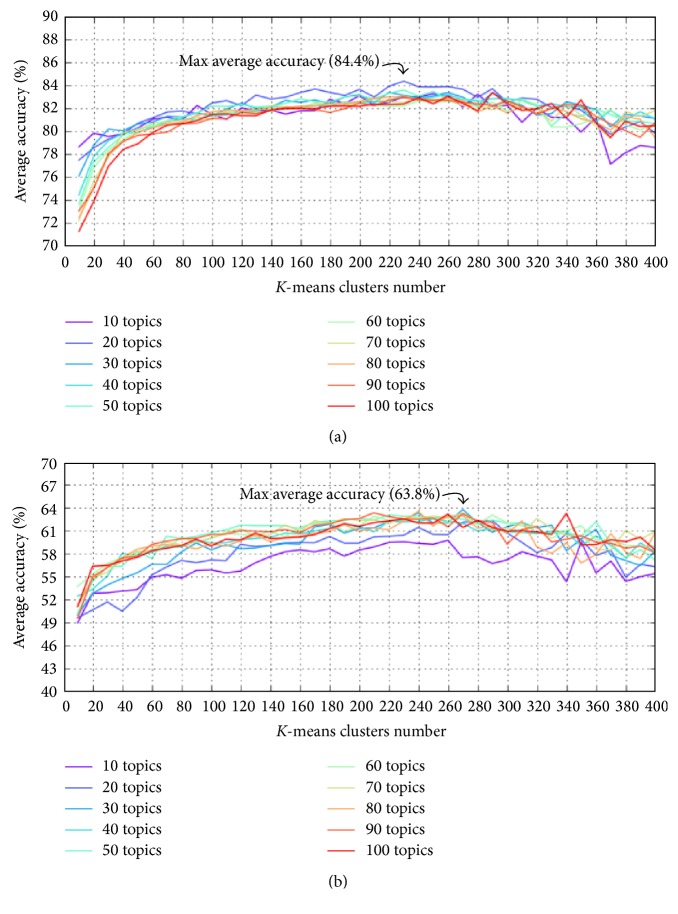
Average accuracy of (a)* FrameDiff_GridBlock_Kmeans* and (b)* MultiCue_GridBlock_Kmeans*, with *K* ranging from 10 to 100 and *c* ranging from 10 to 400.

**Figure 12:**
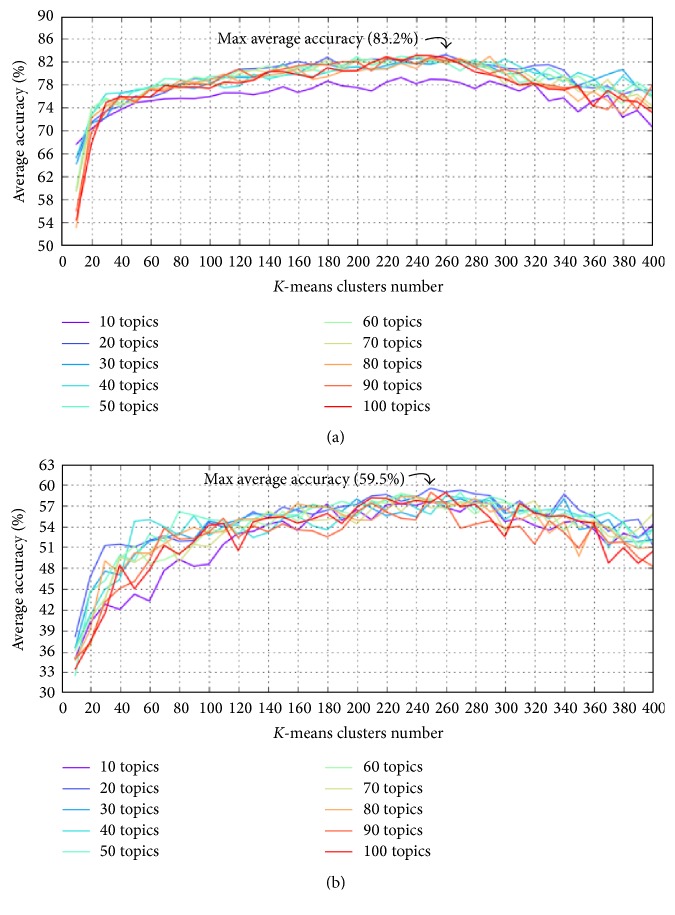
Average accuracy of (a)* FrameDiff_GridIntersect_Kmeans* and (b)* MultiCue_GridIntersect_Kmeans*, with *K* ranging from 10 to 100 and *c* ranging from 10 to 400.

**Figure 13:**
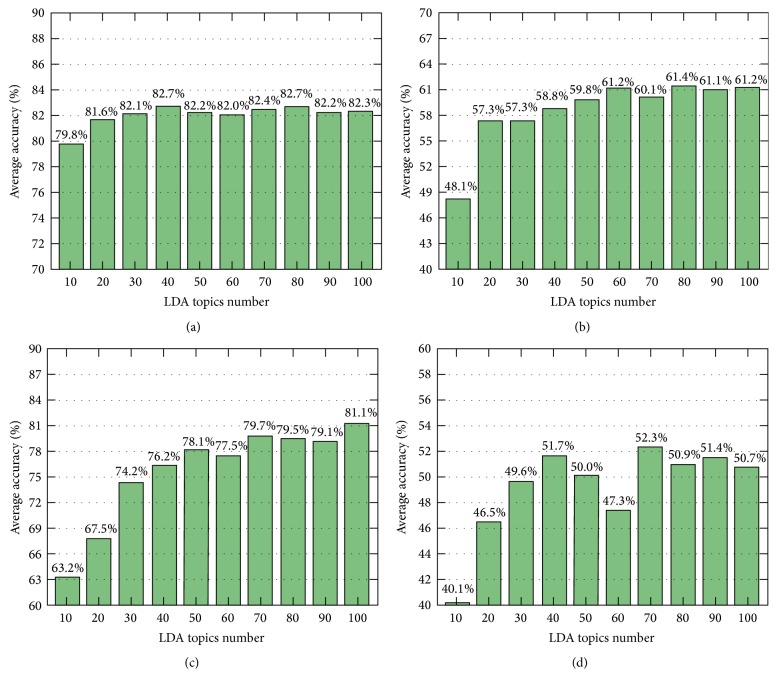
Average accuracy of (a)* FrameDiff_GridBlock_Top*, (b)* MultiCue_GridBlock_Top*, (c)* FrameDiff_GridIntersect_Top*, and (d)* MultiCue_GridIntersect_Top*, with *K* ranging from 10 to 100 and *c* ranging from 10 to 400.

Figures 11, 12, and 13 show the average accuracy of each method when parameters *K* and *c* changed. As described above, while the segmentation results of all methods using *k*-means feature normalization method depend on both parameters *K* and *c*, the results of methods using* Top* depend only on *c*. Therefore, in the figures, we used two different types of figures to show the results.

Figure 15 and Table 7 show a comparison between the maximum average accuracies of different existing methods with different values of topics number *K* and *k*-means clusters number *c*. More specifically, from results of each method (i.e., Figure 11(a)), we select the parameters setting which yielded the best average accuracy (i.e., 84.4% in Figure 11(a)) as the best-suited setting for parameters. The average accuracy which corresponds to the best-suited parameters setting is the maximum average accuracy of the method. The purpose of Figure 15 and Table 7 is to compare proposed methods after fixing parameters to their own best-suited values. Because the* Top* feature normalization method has no parameter *c*, all methods using* Top* are denoted as—in column *c* of the table. In addition, Figure 16 and Table 8 show an example of phase segmentation outputs for a test surgical workflow using our proposed* FrameDiff_GridBlock_Kmeans* method.

**Figure 14:**
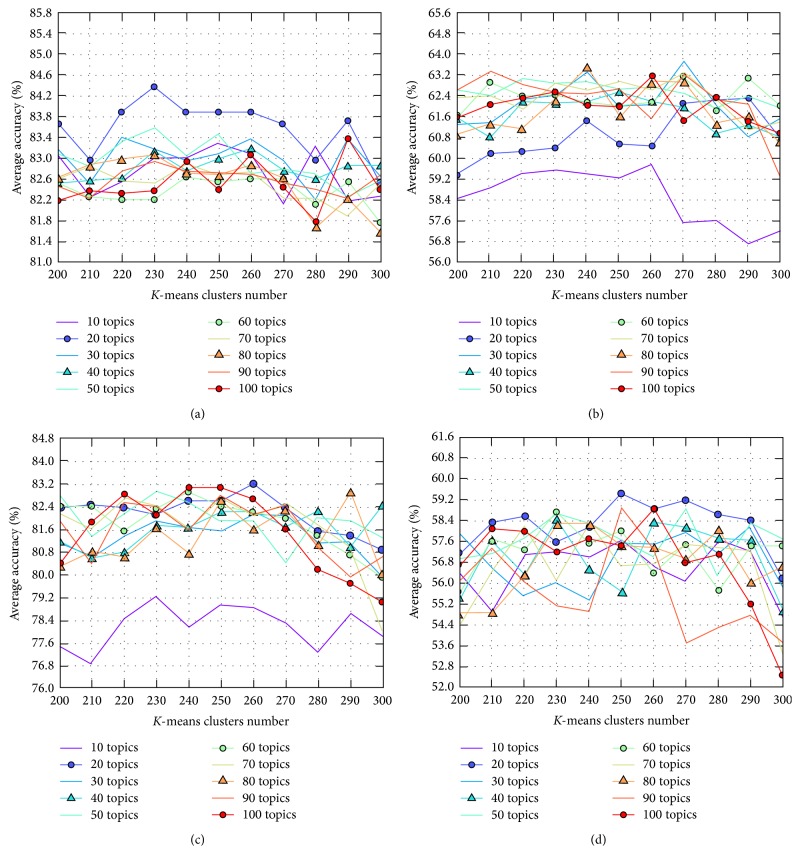
Average accuracy of (a)* FrameDiff_GridBlock_Kmeans*, (b)* MultiCue_GridBlock_Kmeans*, (c)* FrameDiff_GridIntersect_Kmeans*, and (d)* MultiCue_GridIntersect_Kmeans*, with *K* ranging from 10 to 100 and *c* ranging from 200 to 300.

In Figures 11 and 12, we observe that for all numbers of topics, when the number of clusters increased, the phase segmentation results were more accurate; however, the accuracy tended to decrease as the number of clusters grew too large. The best choice for *k*-means clusters in our experiments was therefore between 200 and 300. We also observe that changes in the number of clusters significantly impacted segmentation results, whereas the number of topics yielded nearly equivalent results except for when the number of topics was 10; however, this value of 10 for parameter *K* was too small to express the features of all motions for this surgical workflow.  Figure 14 is similar to Figures 11 and 12 but only shows results when *K* was from 200 to 300 to make these results easier to see.

Table 7 and Figure 13 indicate that the methods using* Top* tended to be more accurate when the number of topics *K* was large; conversely, Table 7 and Figures 11 and 12 indicate that in methods using *k*-means a large enough number of *k*-means clusters *c* and a small number of topics *K* (but not less than 20) yielded better accuracy.

**Figure 15:**
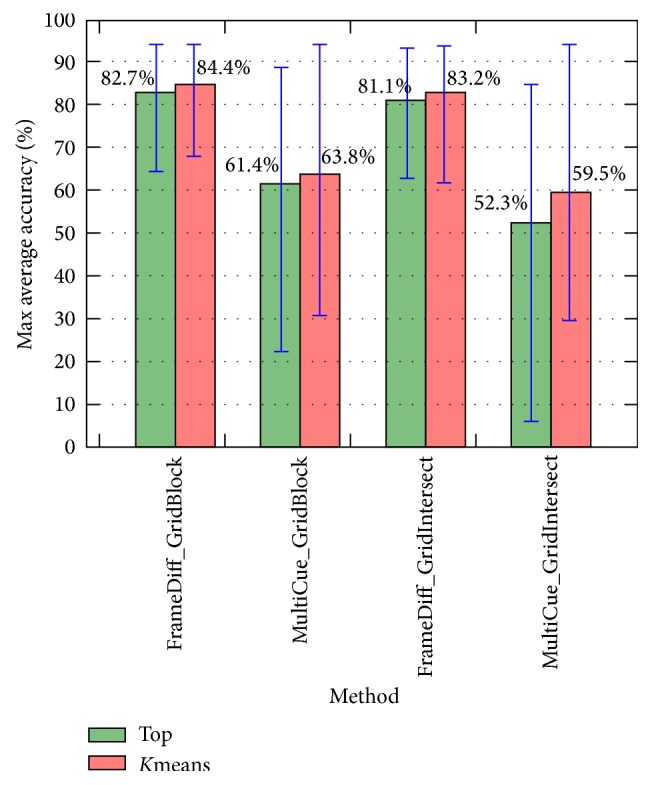
Maximum average accuracy of all methods with *K* ranging from 10 to 100 and *c* ranging from 10 to 400.

**Figure 16:**
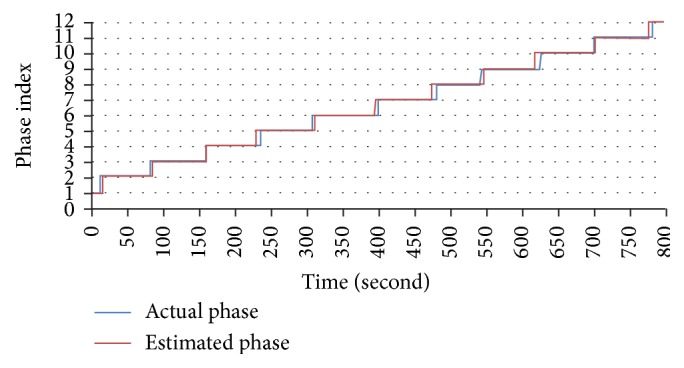
An example of phase segmentation outputs for a test surgical workflow using* FrameDiff_GridBlock_Kmeans* method.

We also observe in Figure 15 that methods using *k*-means were always better than ones using* Top*. Further, the use of* GridBlock* OF extraction yielded higher levels of accuracy than that of* GridIntersect* OF extraction, but the differences here are not entirely clear. We note for these results that* GridIntersect* extracts only OFs at a fixed number of points, namely, at the intersection of vertical and horizontal lines; thus it is easy to lose important motion features. On the other hand,* GridBlock* uses averaged OFs; thus it is able to learn more important movements.

Figure 15 also indicates that using* FrameDiff* to detect the foreground was far better than using* MultiCue* in terms of both maximum average accuracy and error. While* FrameDiff* detects only the contour of moving contexts,* MultiCue* tends to also detect the region inside the contour (see Figure 3), indicating that extracting OFs only at the points connecting foreground and background provides more robust features than at all points within the foreground. Moreover, because* FrameDiff* is a very simple foreground detection method, its execution time is substantially less than that of* MultiCue*. In summary,* FrameDiff* is well-suited in this case.

**Figure 17:**
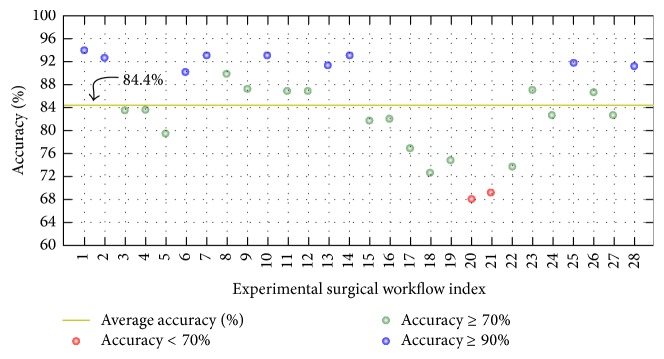
Accuracy in each surgical workflow of* FrameDiff_GridBlock_Kmeans* when *K* was 20 and *c* was 230.

Finally, the maximum average accuracy achieved in our experiments was 84.4% when using* FrameDiff* foreground detection,* GridBlock* OF extraction, and *k*-means feature normalization. The numbers of topics and *k*-means clusters in this case were set to 20 and 230, respectively.  Figure 17 shows details about the accuracy of each of 28 experimental surgical workflows in this case. In the figure, the yellow line indicates the average accuracy, and the blue and green dots indicate surgical workflows with their own accuracy being not less than 70%, while surgical workflows having level of accuracy lower than 70% are shown as red dots. If we select the value of 70% for accuracy as criteria to evaluate all staff, we observe that staff that performed 20th and 21st surgical workflows have low level of experience and performance; such staff should therefore be trained more. In case of danger, unusual events may have occurred in such surgical workflows, and the staff must be given warnings in all such situations to avoid medical errors, which directly impact patient safety. On the other hand, all staff that performed surgical workflows with their own accuracy being not less than 90% can be seen as experts. In summary, these experiments demonstrate that our proposed methods are able to achieve very promising results. Moreover, our present work can be further used for training medical staff, issuing warnings during surgical workflow, and so forth.

#### 4.2.2. Calculation Time

Table 9 shows the average calculation time of all methods for a test surgical workflow with the average workflow length being 12.8 minutes (18432 frames). We observe that, although using* FrameDiff_GridBlock* methods was slightly better than using* FrameDiff_GridIntersect* methods in terms of accuracy (see Figure 15),* FrameDiff_GridIntersect* methods were far faster than* FrameDiff_GridBlock* methods, with just 4.0-minute calculation time for a 12.8-minute workflow.* FrameDiff_GridBlock* methods had a speed of 55.7 minutes per 12.8-minute workflow, which is an acceptable speed. Unfortunately, although* MultiCue_GridIntersect* methods were very fast, their accuracies were worst in 8 methods.* MultiCue_GridBlock* methods were not good in both terms of calculation time and accuracy.

In addition, Table 10 shows more details about average calculation time of each step in all methods for a test surgical workflow. The table indicates that, in total time spent to segment phases of the test workflow, almost time was used to detect foreground and extract OFs. LDA, feature normalization, and HMM took very small time and they were trivial. In foreground detection step, we observe that* FrameDiff* was about 4 times faster than* MultiCue*. This can be explained by the fact that* FrameDiff* which only calculates the absolute difference between two consecutive frames to detect foreground is a very simple approach. On the other hand,* MultiCue* uses more complex approaches (i.e., codebook) to obtain characteristics of pixel texture, pixel color, and local image appearance for detecting foreground. In OF extraction step, we observe that* GridBlock* was far slower than* GridIntersect*. It can be explained because* GridBlock* calculates OFs at all foreground pixels in each block, while* GridIntersect* calculates them at only the intersection pixels between lines (see Section 3.2).

## 5. Conclusions

In this paper, we described new methods that use LDA and HMM approaches to automatically segment phases of a specified surgical workflow. The input to these methods consists of multiple videos acquired from multiple synchronized cameras. After all processes, including foreground detection, OF extraction, and topics modeling, using both LDA and HMM construction are completed, we are able to estimate the appropriate phase at a given time point of the workflow. Our proposed methods accomplished the following:

- They retained a high accuracy even for large datasets. In our experiments, we showed the robustness of our methods as having a maximum average accuracy of 84.4% for a dataset consisting of up to 28 surgical workflows with seven different participants.
- They ran at a high speed (in cases of* FrameDiff_GridIntersect*,* MultiCue_GridIntersect*, and* FrameDiff_GridBlock*).
- They used camera sensor, which not only is easy to set up, but also has a substantial advantage as compared to other sensors; that is, despite the existence of cameras in the operating room, all staff do not notice the presence of such cameras; thus there is no risk for the surgical workflow to be negatively impacted due to their existence.
- They used OF extraction for acquiring motion features of all general working contexts; thus not only medical staff motions, but also information about equipment and materials being used is covered.

For our future work, we plan to recognize the use of surgical instruments and improve upon our phase segmentation algorithm to obtain higher levels of performance. As a next step, we will further develop an automatic moving camera system, in which cameras can automatically move on the wall and ceiling to positions that can acquire the most meaningful and important information, via human and object tracking algorithms. This means that, in case of more complex and realistic operating room, namely, if there are multiple staff performing surgery, our system can automatically separate targets for each camera, and each camera can then track, focus on, and acquire motion information from only one staff. As a result, the phase segmentation becomes more accurate. Next, we plan to extend our proposed system for constructing

- unusual event detection system to reduce medical errors,
- medical education support system to train larger numbers of staff,
- automated system that can perform daily medical work in place of staff,
- surgical decision-making system to recommend or prompt the appropriate actions during surgical workflow,
- documentation generation system to automatically generate documents, reports, and so forth, after each surgical workflow.

## Figures and Tables

**Table 1:** Foreground detection methods.

Abbreviation	Method name	Authors
FrameDiff	Frame difference	—
MultiCue	Multicue	[17]

**Table 2:** Abbreviations for OF extraction methods.

Abbreviation	Method name
GridIntersect	Grid intersection
GridBlock	Grid block

**Table 3:** Combined methods constructed from all foreground detection and OF extraction methods.

Combined abbreviation	Foreground detection	OF extraction
FrameDiff_GridIntersect	FrameDiff	GridIntersect
MultiCue_GridIntersect	MultiCue	GridIntersect
FrameDiff_GridBlock	FrameDiff	GridBlock
MultiCue_GridBlock	MultiCue	GridBlock

**Table 4:** Abbreviations for feature normalization methods.

Abbreviation	Method name
Top	Top as One, Another as Zero
*K*means	*K*-means

**Table 5:** The 12 phases of the cholecystectomy surgical workflow.

Phase index	Phase name
1	Materials preparation
2	CO2 inflation
3	Trocar insertion
4	Dissection
5	Clipping cutting
6	Gallbladder detaching
7	Liver bed coagulation
8	Packaging of gallbladder
9	External cleaning
10	Trocar retraction
11	Abdominal suturing
12	Materials return

**Table 6:** Combined methods constructed from all foreground detection, OF extraction, and feature normalization methods.

Combined abbreviation	Foreground detection and OF extraction	Feature normalization
FrameDiff_GridIntersect_Top	FrameDiff_GridIntersect	Top
MultiCue_GridIntersect_Top	MultiCue_GridIntersect	Top
FrameDiff_GridBlock_Top	FrameDiff_GridBlock	Top
MultiCue_GridBlock_Top	MultiCue_GridBlock	Top
FrameDiff_GridIntersect_*K*means	FrameDiff_GridIntersect	*K*means
MultiCue_GridIntersect_*K*means	MultiCue_GridIntersect	*K*means
FrameDiff_GridBlock_*K*means	FrameDiff_GridBlock	*K*means
MultiCue_GridBlock_*K*means	MultiCue_GridBlock	*K*means

**Table 7:** Maximum average accuracy of all methods with *K* ranging from 10 to 100 and *c* ranging from 10 to 400.

Method	Max average accuracy	*K*	*c*
FrameDiff_GridBlock_Top	82.7%	80	—
FrameDiff_GridBlock_*K*means	84.4%	20	230
MultiCue_GridBlock_Top	61.4%	80	—
MultiCue_GridBlock_*K*means	63.8%	30	270
FrameDiff_GridIntersect_Top	81.1%	100	—
FrameDiff_GridIntersect_*K*means	83.2%	20	260
MultiCue_GridIntersect_Top	52.3%	70	—
MultiCue_GridIntersect_*K*means	59.5%	20	250

**Table 8:** An example of phase segmentation outputs for a test surgical workflow using *FrameDiff_GridBlock_Kmeans* method.

Phase index	Start time (seconds)	Actual phase length (seconds)	Deviation (seconds)
1	1	13	0
2	14	68	3
3	82	80	5
4	162	76	0
5	238	72	6
6	310	93	2
7	403	81	6
8	484	61	8
9	545	83	5
10	628	76	7
11	704	81	2
12	785	15	5

Average		66.6	4.1

**Table 9:** Average calculation time of all methods for a test surgical workflow (12.8 minutes of length).

Method	Average calculation time (minutes)
FrameDiff_GridBlock_Top	55.7
FrameDiff_GridBlock_*K*means	55.7
MultiCue_GridBlock_Top	334.3
MultiCue_GridBlock_*K*means	334.3
FrameDiff_GridIntersect_Top	4.0
FrameDiff_GridIntersect_*K*means	4.0
MultiCue_GridIntersect_Top	9.3
MultiCue_GridIntersect_*K*means	9.3

**Table 10:** Average calculation time of each step in all methods for a test surgical workflow (12.8 minutes of length).

Method	Foreground detection (milliseconds per frame)	OF extraction (milliseconds per frame)	LDA (seconds per workflow)	Feature normalization (milliseconds per workflow)	HMM (milliseconds per workflow)
FrameDiff_GridBlock_Top	4.7	176.3	4.7	61.0	7.6
FrameDiff_GridBlock_*K*means	4.7	176.3	4.7	57.0	23.1
MultiCue_GridBlock_Top	17.7	1070.0	10.1	79.0	9.7
MultiCue_GridBlock_*K*means	17.7	1070.0	10.1	74.0	24.6
FrameDiff_GridIntersect_Top	4.7	8.4	0.9	54.0	14.2
FrameDiff_GridIntersect_*K*means	4.7	8.4	0.9	57.0	20.5
MultiCue_GridIntersect_Top	17.7	12.2	6.3	71.0	12.6
MultiCue_GridIntersect_*K*means	17.7	12.2	6.3	69.0	24.2
